# Modulation of the Lower Critical Solution Temperature of Thermoresponsive Poly(*N*-vinylcaprolactam) Utilizing Hydrophilic and Hydrophobic Monomers

**DOI:** 10.3390/polym15071595

**Published:** 2023-03-23

**Authors:** Elaine Halligan, Shuo Zhuo, Declan Mary Colbert, Mohamad Alsaadi, Billy Shu Hieng Tie, Gilberto S. N. Bezerra, Gavin Keane, Luke M. Geever

**Affiliations:** 1 Polymer, Recycling, Industrial, Sustainability and Manufacturing (PRISM) Center, Technological University of the Shannon: Midlands Midwest, Dublin Road, Athlone, N37 HD68 Co. Westmeath, Ireland; 2 CONFIRM Centre for Smart Manufacturing, University of Limerick, V94 C928 Co. Limerick, Ireland; 3 Centre for Industrial Service & Design, Technological University of the Shannon: Midlands Midwest, Dublin Road, Athlone, N37 HD68 Co. Westmeath, Ireland; 4 Applied Polymer Technologies Gateway, Material Research Institute, Technological University of the Shannon: Midlands Midwest, Dublin Road, Athlone, N37 HD68 Co. Westmeath, Ireland

**Keywords:** *N*-vinylcaprolactam, copolymers, photopolymerisation, lower critical solution temperature, hydrogel

## Abstract

Four-dimensional printing is primarily based on the concept of 3D printing technology. However, it requires additional stimulus and stimulus-responsive materials. Poly-*N*-vinylcaprolactam is a temperature-sensitive polymer. Unique characteristics of poly-*N*-vinylcaprolactam -based hydrogels offer the possibility of employing them in 4D printing. The main aim of this study is to alter the phase transition temperature of poly-*N*-vinylcaprolactam hydrogels. This research focuses primarily on incorporating two additional monomers with poly-*N*-vinylcaprolactam: Vinylacetate and *N*-vinylpyrrolidone. This work contributes to this growing area of research by altering (increasing and decreasing) the lower critical solution temperature of *N*-vinylcaprolactam through photopolymerisation. Poly-*N*-vinylcaprolactam exhibits a lower critical solution temperature close to the physiological temperature range of 34–37 °C. The copolymers were analysed using various characterisation techniques, such as FTIR, DSC, and UV-spectrometry. The main findings show that the inclusion of *N*-vinylpyrrolidone into poly-*N*-vinylcaprolactam increased the lower critical solution temperature above the physiological temperature. By incorporating vinylacetate, the lower critical solution temperature dropped to 21 °C, allowing for potential self-assembly of 4D-printed objects at room temperature. In this case, altering the lower critical solution temperature of the material can potentially permit the transformation of the 4D-printed object at a particular temperature.

## 1. Introduction

Smart hydrogels are materials composed mostly of water, which maintains a three-dimensional (3D) hierarchical structure of polymer chains, which provide them with a gel-like consistency and allow the hydrogels to respond to several external stimuli to induce a response [1]. Such stimuli may include pH, salt composition, solvent composition, and temperature [2]. Hydrogels possess many advantages, such as their high hydrophilicity, ability to control the release of active pharmaceutical ingredients (API), biocompatible nature, and many others [3]. Hydrogels can be produced through relatively simple procedure [4] while also using inexpensive materials [5,6], which is yet another significant advantage of the use of hydrogels. The main drawbacks regarding the use of hydrogels are their comparatively poor mechanical properties when in their swollen state, rapid release of API, poor bacterial barrier properties, gas and water vapor permeability, and non-uniformity of loaded hydrophobic API [7]. Often, hydrogels are weak and brittle, which impedes their use in applications where a high degree of strength is required. This may be overcome, however, through the addition of a stiff shape-memory polymer [8]. The classification of hydrogels is dependent on several criteria, such as their physical properties, the nature of swelling, the method of preparation, origin, ionic charges, rate of biodegradation, as well as the nature of crosslinking [9]. Broadly speaking, this classification can be divided into either permanent or reversible gels. Permanent gels are those that possess covalently crosslinked networks whereas reversible gels possess networks composed of molecular entanglements and/or secondary interactions, such as hydrogen bonding (H-bonding), ionic bonding, or hydrophobic interactions [10]. Hydrogels have drawn great attention for use across a wide variety of applications that may be applied to agriculture, wound healing, sealing, hygienic products, diagnostic, separation of biomolecules or cells, tissue engineering, and regenerative medicines, biomedical applications, drug delivery systems, food additives, pharmaceuticals, and biosensors [8]. The attention toward hydrogels within biomedical applications is attractive due to their biocompatibility and the similarity of their physical properties to natural tissue [11]. Recently, Haleem et al. (2021) described the fabrication of hydrogels at a negative temperature, resulting in macroporous hydrogels named cryogels. Cryogels have gained importance due to their large pore size, rough surface area, and fast swelling in the field of catalysis, oil/water, separation, energy production, and solar heating water evaporation [12,13].

Poly-*N*-vinylcaprolactam (PNVCL) is a synthetic hydrogel-forming polymer. It is a non-ionic, water-soluble, and thermoresponsive polymer with the capability of transitioning from a liquid state to a gel state when exposed to temperatures above its lower critical solution temperature (LCST), which is commonly reported as being close to the physiological temperature range 34–37 °C [14,15,16,17,18,19]. Overall, PNVCL is an inexpensive and highly used polymer due to its low cytotoxicity, which enables the polymer to be employed in the biomedical field for applications, such as wound healing, drug delivery, and tissue engineering [20]. As mentioned by Frost et al. (2017), PNVCL can undergo abrupt physical or chemical changes depending on the response to external environmental stimuli, such as temperature, pH, electric, and magnetic fields [21]. In the last ten years, much more research has been carried out on the synthesis and characterisation of novel temperature-responsive polymers, such as PNVCL, to alter the LCST, and thus, transitions in the physiological range can be achieved. Yu et al. (2013), focused on the alteration of LCST through the incorporation of *N*-vinylpyrrolidone into PNVCL blocks. This copolymerisation allowed for a reduction in LCST to match body temperature, thereby improving its future potential use in the biomedical field [14]. More recent evidence highlights the effect of acetic acid (AA) on PNVCL LCST for biomedical applications. In the aforementioned investigation, Dalton et al. (2018) concluded that the LCST of PNVCL/AA solutions increased with respect to an increase in AA concentration due to the increased hydrophilicity provided by the AA [22]. These unique characteristics of the PNVCL-based hydrogels offer the possibility of employing them in four-dimensional (4D) printing.

Four-dimensional printing is the next evolution in 3D printing. Whereas 3D printing is capable of producing a static physical part, 4D printing allows for the production of a part with the capability of reacting to external stimuli (such as air, heat, or other chemical reactions) to instigate dynamic movement [23,24]. The interest in 4D printing has been gaining traction since the publication of the first 4D printing-based research paper in 2013 [25]. The focus of the afforementioned paper was the development of printed active composites (PACs) as laminated sheets, the morphology of which could be transformed into a complex structure and subsequently returned to its original conformation upon exposure to elevated temperature [26]. Significant interest lies in utilising 4D printing in the biomedical field, especially in tissue engineering and targeted drug delivery [27,28]. The smart materials employed in the 4D printing process may contain API and subsequently release it when the conditions of the target environment are sufficient as activating stimuli. Melocchi et al. (2019) have demonstrated 4D-printed PVA-based expandable drug delivery systems (EDDSs), which would respond to the conditions of the gastrointestinal tract to allow for retention and subsequent drug release [29]. Zhao et al. (2021) demonstrated self-folding GelMA tubes for tissue engineering applications. Produced as bilayer films, when exposed to aqueous environments, the material self-assembles into tubes with a diameter of 6 mm with a controlled release of heparin following [30].

Modulation of the transition temperature can be very useful in the process of 4D-printing technology, as temperature is the most common stimulus used to induce shape changes in 4D materials [31]. One of the most crucial factors affecting the design of 4D-printed hydrogels is the LCST. The LCST of PNVCL and PNVCL-based copolymers may be affected by the composition of the copolymers or the concentration in aqueous media. In this work, the authors wish to examine the alteration of the phase transition temperature of PNVCL using photopolymerisation to graft copolymers of PNVCL and either vinylacetate (VAc) or *N*-vinylpyrrolidone (NVP). As such, this study lays the groundwork for 4D printing with PNVCL and PNVCL-based copolymers to provide essential insights into the alteration of the phase transition temperature to allow the 4D-printed object to transform at a specific temperature. 

## 2. Materials and Methods

### 2.1. Materials

*N*-vinylcaprolactam (NVCL) was used as a main material with a molecular weight of 139.19 g/mol and a storage temperature from 2 to 8 °C and was supplied by Sigma Aldrich, Co. Dublin, Ireland. The photoinitiator, 4-(2hydroxyethoxy) phenyl-(2-hydroxy-2-propyl) ketone (Irgacure^®^ 2959 Ciba Corp, New York, NY, USA), was obtained from Ciba Specialty Chemicals. Vinylacetate (VAc) and *N*-vinylpyrrolidone (NVP) with a molecular weight of 86.09 g/mol and 111.14 g/mol were obtained from Sigma Aldrich, Ireland. The chemical structure of these materials is presented in Table 1.

### 2.2. Synthesis of Thermosensitive PNVCL Hydrogel

PNVCL, PNVCL/VAc, and PNVCL/NVP were photopolymerised using the Dr. Gröbel UV-Elektronik GmbH, Ettlingen, Germany, UV curing system. This curing system consists of an irradiation chamber allowing for controlled radiation with 20 UV tubes, provides a light spectrum wavelength in the range between 315 and 400 nm at an average intensity of 10–13.5 mW/cm^2^. The mixtures were prepared using specific amounts of VAc/NVP and NVCL. Irgacure^®^ 2959 photoinitiator was combined with the mixtures at 0.1 wt% to ensure the consistency of samples. The batches were placed in a 25-mL glass beaker and were allowed to mix using a magnetic stirrer for 20 min. The disc-like silicone mould was then placed horizontally under the UV tubes; then, the solutions were pipetted into the mould. Following this, the solutions were allowed to UV cure for 30 min. After 15 min of curing, samples were turned to ensure that the entire surface area of each sample received the same intensity of light radiation during the photopolymerisation process. After successful curing, the samples were dried in a vacuum oven at 50 °C for 24 h before use. The sample formulations of all produced PNVCL-based samples are shown in Table 2.

### 2.3. Attenuated Total Reflectance Fourier Transform Infrared Spectroscopy

Attenuated total reflectance Fourier transform infrared spectroscopy (ATR-FTIR) was conducted using a Perkin Elmer Spectrum One FT-IR spectrometer (C-001), Waltham, MA, United States fitted with a universal ATR sampling accessory. All data were recorded at room temperature at approximately 22 °C, in the spectral range of 4000–650 cm^−1^, utilising a fixed universal compression load of 75 N and a 4-scan per sample cycle. Subsequent analysis was performed using Perkin Elmer spectrum software (SpectrumTM 10).

### 2.4. Differential Scanning Calorimetry

To observe the thermal transitions of the utilised materials differential scanning calorimetry (DSC) was employed. A differential scanning calorimeter (TA Instrument DSC 2920 Modulated DSC, New Castle, DE, USA) was used. Samples were prepared by weighing an appropriate amount of sample ranging from 8–12 mg using a Sartorius balance with a resolution of 0.01 mg. The samples were then placed in hermetically sealed aluminium pans, which were subsequently crimped before testing. A similarly prepared empty pan was used as a reference. The instrument was fully calibrated using indium as the standard. Scans were performed at a rate of 10 °C/min from 20–200 °C. Volatiles were removed from the purging head using nitrogen gas at a flow rate of 30 mL/min.

### 2.5. Phase Transition Determination

#### 2.5.1. Cloud Point Analysis

Cloud point measurement was conducted to investigate the samples transition temperature, deemed the cloud point temperature (T_cp_). This test involved using a Memmert water bath (Memmert, Schwabach, Germany) in which the temperature was increased manually at a rate of ≤1 °C per 2 min. Based on the literature, the samples were prepared at two concentrations, 5 and 10 wt% [22], and placed into sealed 75-mm glass tubes. For the sample of 5 wt% concentration, 0.5 g of a sample and 9.5 g of deionised water (d.H_2_O) were added to each of the sealed tubes. To obtain the 10 wt% concentration, 1 g of a sample and 9 g of deionised water were added to each tube. The resulting LCST was recorded at the specific temperature at which the samples began to show initial signs of turbidity. To ensure the accuracy of the water bath’s temperature, a thermometer with an accuracy of ±0.2 °C was placed in the water bath. This test was carried out in duplicate, and photographs were taken of the samples to visualise the contrast between the samples that were both above and below the phase transition temperature.

#### 2.5.2. UV-Vis Spectrometry Analysis

UV-vis spectrometry was performed by dissolving two known concentrations of polymer (5 and 10 wt%) in deionised water. The aqueous solutions were maintained at room temperature (~22 °C) for 24 h to reach an ambient equilibrium. The UV-vis spectrometer (Hitachi BioTec), (Biotek Synergy, Britanya, CA, USA) fitted with a temperature ramp heating system, monitored the optical absorbance at 500 nm for each polymer solution as a function of temperature. A heating rate of 1 °C/min from ambient temperature to 45 °C was employed for this analysis.

### 2.6. Swelling Analysis

Initially, the dry mass of the polymerised samples was measured using a Sartorius balance, (Sartorius, Goettingen, Germany) with a resolution of 1 × 10^−5^ and denoted as *W_d_*. The samples were then placed into glass Petri dishes containing 25 mL of distilled water (pH = 7.1). Samples were tested under ambient temperature conditions. After specific predetermined time intervals, the samples were removed from the Petri dishes and blotted dry of excess surface water by use of filter paper, and the wet weight was recorded as *W_t_*. All samples were performed in triplicate. The swelling ratio was subsequently calculated using Equation (1).

*Swelling Ratio* (%) = ((*W_t_* − *W_d_*)/*W_d_*) × 100 (1)

where *W_t_* is the weight of the gel at a predetermined time, and *W_d_* is the dry mass weight of the polymer.

### 2.7. Gelation Determination

A tube inversion method determined the phase transition from the sol–gel of the copolymers in an aqueous solution. The aqueous polymer solutions were dissolved in water at two concentrations (5 and 10 wt%) and placed in glass test tubes with a diameter of 10 mm. The test tubes were maintained at a constant temperature (20 °C) for 15 min as the temperature was increased manually (±1 °C) in a Memmert water bath prior to inversion. By inverting the tubes horizontally, the sol–gel transition was determined. This experiment was carried out at 1 °C/min intervals. The samples were placed in an ice-water bath prior to switching to the next temperature and made homogenous. The sol–gel transition temperature was considered to be the temperature at which the polymer solution did not flow when the tube was inverted.

## 3. Results and Discussion

### 3.1. Photopolymerisation of Xerogels

Photopolymerisation is a method of polymerisation where visable or UV light is used to form in situ cross-linkages to polymerise a sample [32]. Photopolymerisation offers numerous advantages over other polymerisation techniques, such as minimal heat production during the process and being a comparatively rapid process; polymerisation occurs from less than a second to several minutes [33]. This technique was chosen as it utilises the same mechanism as that of stereolithography (SLA) 3D printing, and so the produced samples would act as a proxy-precursor of 3D-printed samples.

**Figure 1:**
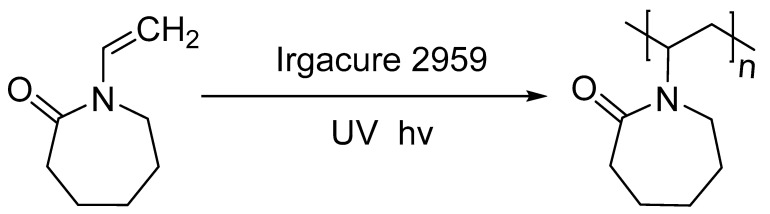
Schematic representation of the photopolymerisation process of NVCL to PNVCL in the presence of Irgacure^®^ 2959 [36].

The homopolymers and copolymers, PNVCL/NVP and PNVCL/VAc, were photopolymerised in the presence of 0.1 wt% Irgacure^®^ 2959 photoinitiator. A minimal amount of photoinitiator was used in an attempt to limit the cytotoxicity of samples. Reports regarding Irgacure^®^ 2959 have stated that this photoinitiator is the most frequently used photoinitiator in aqueous photocuring systems. When exposed to UV light, Irgacure^®^ can decompose, thereby generating free radicals. These free radicals in turn initiate the polymerisation process of the monomers. The reaction mechanism of polymerising the NVCL monomer is shown in Figure 1.

**Figure 2:**
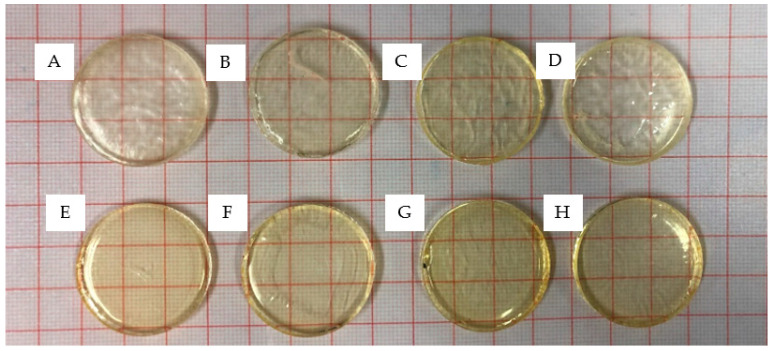
PNVCL xerogels fabricated with varying proportions of VAc/NVP with 0.1 wt% Irgacure^®^ 2959 initiator: (**A**) B1: P(NVCL90-VAc10), (**B**) B2: P(NVCL80-VAc20), (**C**) B3: P(NVCL70VAc30), (**D**) B4:P(NVCL60-VAc40), (**E**) C1:P(NVCL90-NVP10), (**F**) C2:P(NVCL80-NVP20), (**G**) C3:P(NVCL70NVP30), and (**H**) C4:P(NVCL60-NVP40).

**Figure 3:**
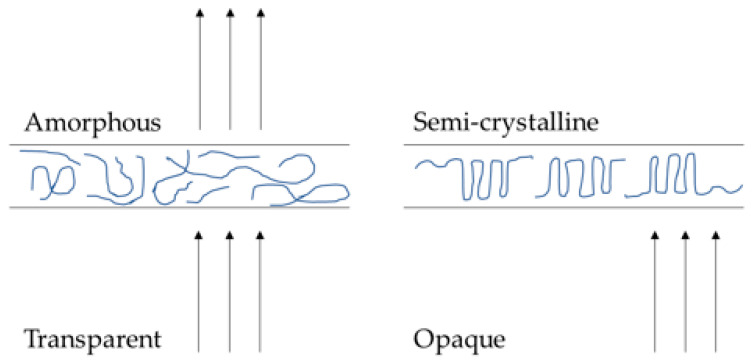
The more highly-organised polymeric structure found within semi-crystalline polymers can disrupt the transmission of light rays, rendering the material opaque.

A silicone mould with disc-shaped cavities was used for the preparation of test samples (formulations in Table 2) and dried for 24 h in a vacuum oven after removal from the silicone mould. As shown in Figure 2, all samples cured well and maintained structural integrity post-removal. The discs were glass-like with a high degree of transparency. In semi-crystalline polymers, light can scatter at the boundaries between the crystalline and amorphous regions. This in turn leads to less light transmission through the polymer, which results in an opaque sample. The transparent nature of the disc samples produced herein could be inferred as an indication that the samples were amorphous with randomly distributed polymer chains with no distinct boundaries [34,35]. Figure 3 displays the highly-organised polymeric structure found within semi-crystalline polymers that can disrupt the transmission of light rays, rendering the material opaque.

### 3.2. Attenuated Total Reflectance Fourier Transform Infrared Spectroscopy

**Figure 4:**
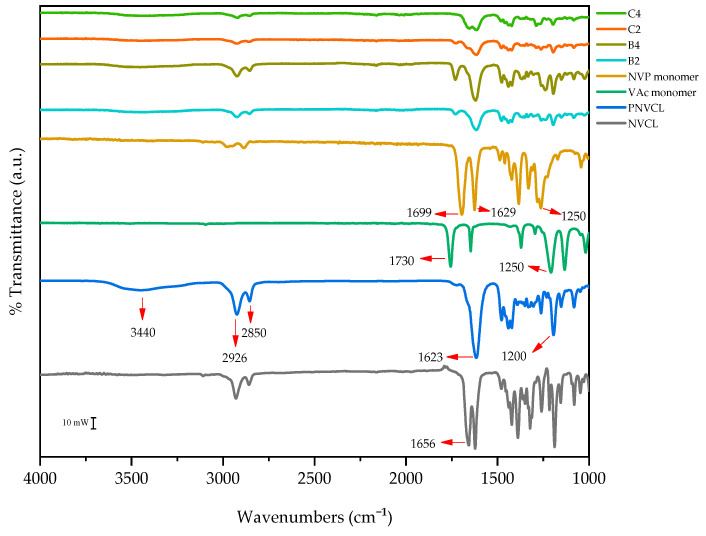
FTIR spectra of (**top**–**bottom**) P(NVCL60-NVP40), P(NVCL80-NVP20), P(NVCL60-VAc40), P(NVCL80-VAc20), NVP monomer, VAc monomer, PNVCL, and NVCL.

ATR-FTIR was used to observe the variances in the chemical structure of monomers and photopolymerised samples. When a sample is exposed to IR radiation, a proportion will be absorbed by the material while another proportion will pass through the samples (transmission). Different functional groups and chemical bonds will absorb radiation at different wavelengths (absorption bands), which allows for the identification of the functional groups within a sample. In the context of this research, ATR-FTIR was employed to distinguish between the chemical bonds found in the monomers (VAc, NVCL, and NVP) and polymerised samples (A1, B2, B4, C3, and C4). The resultant IR spectra and assignment of absorption bands to functional groups are displayed in Figure 4 and Table 3, respectively.

The monomers displayed characteristic absorption bands at 1730 cm^−1^ (VAc), 1656 cm^−1^ (NVCL), 1640 cm^−1^, and 2800 cm^−1^ (NVP). These absorption bands were assigned to the stretching vibration of the carbonyl (C=O) functional group of the VAc monomer and the stretching vibration of the C=C for the NVCL monomer. Finally, for NVP, the assignment was for the stretching vibration of the C=O group in the amide functional group and the stretching vibration of the C-H group.

For the homopolymer PNVCL, there was an appearance of absorption bands at 1623 cm^−1^, 3440 cm^−1^, and 2926 cm^−1^. These were subsequently assigned as stretching vibration of the C=O group in the amide functional group, stretching vibration of the O-H functional group, and stretching vibration of the C-H functional group, respectively. All NVCL-based samples displayed the aliphatic C-H band. The caprolactam ring was recognised at 2926 cm^−1^ and 2850 cm^−1^. The peak at 1623 cm^−1^ is deemed the amide-1 band and is characteristic of all amides. The specific position of this band is dependent on several criteria, including the physical state of the compound and the degree of H-bonding within the sample [17,37]. The PNVCL homopolymer displayed a broad band at ~3440 cm^−1^ relating to the O-H stretching. The appearance of this broad band is due to PNVCL’s ability to absorb moisture and can be associated with the polymer’s hydrophilic character.

Variation in the IR spectra between samples containing the same constituents is generally indicative of a chemical reaction occurring, thus causing a shift in the chemical structure. This alteration in spectra is evident in Figure 4 for the polymerised samples of PNVCL and VAc. Post-polymerisation, the samples of PNVCL/VAc display a new peak not characteristic of the homopolymer, thus indicating the copolymerisation had successfully occurred. This peak is characteristic of the aliphatic ester group of the Vac, and it can be seen that as the proportion of VAc in the formulation decreases (from 40% to 20%), so too does the intensity of the peak, similar to the findings reported in the literature [34,38]. Similar results were shown in the spectra of PNVCL/NVP copolymers. In the spectrum of the NVP monomer, there is a distinct peak at 1629 cm^−1^ attributed to C=C stretching. As shown in the spectra of the resultant copolymer, however, these peaks disappear, again indicative of successful polymerisation. These findings align with other studies involving the successful polymerisation of PNVCL [39,40].

### 3.3. Differential Scanning Calorimetry

DSC is a form of thermal analysis used to observe the materials’ thermal transitions, heat capacity, and response to a change in temperature. The DSC analysis was conducted to determine the effect of the VAc and NVP monomers on the thermal properties of PNVCL hydrogels. PNVCL is an amorphous polymer with a reported glass transition temperature (T_g_) of 147 °C [41,42,43]. It is also well-documented that the T_g_ of PNVCL may be influenced by such factors as disparity, molar mass, purity, and the presence of moisture in the sample [41,42,43,44].

**Figure 5:**
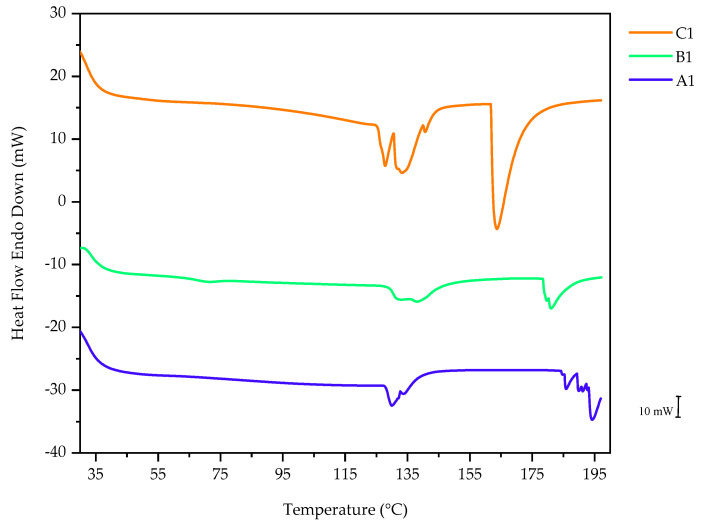
Representative thermograph displaying an overlay of PNVCL, P(NVCL-VAc10), and P(NVCL-NVP10) polymers.

As shown in Figure 5, one broad transition was observed between 60–180 °C for all NVCL-based polymers and was identified as the T_g_ of the PNVCL polymer. For hydrogels to be utilised as delivery systems for API, the T_g_ is an important criterion to consider as, at temperature <T_g_, the diffusion rates will be reduced, while at temperature >T_g_, the diffusion rates will accelerate. Above the T_g_, polymer dissolution occurs along with the formation of a gel layer at the dissolving interface. At this interface, there is polymer chain disentanglement and subsequent diffusion into the surrounding media. Below the T_g_, however, the gel layer thickness is reduced, and the dissolution mechanism is altered from chain disentanglement to an eruption process wherein small blocks of polymer are released [45].

The DSC of A1 featured an endothermic peak at 129 °C. The DSC of B1 exhibited a T_g_ at 70.99 °C. It was observed that the T_g_ of NVCL-based copolymers was dependent on the incorporation level of the monomer. The DSC of B3 exhibited a T_g_ at 63.07 °C. Hence, increasing the concentration of the VAc monomer led to a decrease in the T_g_ value. This behaviour indicates that incorporating VAc has a plasticising effect on PNVCL. This is likely due to the low molecular weight (86.09 g/mol) of VAc, allowing for greater flexibility in the polymer’s chains, resulting in lowered T_g_ values.

### 3.4. Phase Transition Determination

The LCST of temperature-responsive polymers plays a crucial role in designing smart 4D objects. The most important factors affecting the phase transition of NVCL-based hydrogels are copolymer composition and concentrations in aqueous media. The LCST of PNVCL has been reported to be within the range of 25–50 °C [46]. Though the LCST of this polymer has been shown within this broad range, it is often reported as having a well-defined LCST of 32 °C [20,47,48,49]. In order to observe the phase transition of NVCL-based polymers three different techniques were employed to determine the LCST: cloud point analysis, UV-spectroscopy, and gelation determination. Through the implementation of these three techniques, common trends were established.

#### 3.4.1. Cloud Point Analysis

The phase transition temperature of PNVCL, PNVCL/VAc, and PNVCL/NVP at different polymer concentrations was determined by undertaking a visual cloud point analysis. The phase transition temperature was recorded when the sample initially began to show signs of turbidity [22], with these temperatures outlined in Table 4. At ambient temperature, the aqueous polymer solution was transparent due to the effect of the hydrophobic chains. The polymers precipitated gradually, resulting in the solution becoming opaque upon the temperature increase. At a specific temperature, the water becomes a poor solvent for the polymer, possibly attributable to the new and less polar polymer conformation. Additionally, the H-bonds were broken by the growing internal energy, resulting in increased entropy in the system. In order to maintain the balance of entropy, the prevalence of hydrophobic molecules tended to aggregate, leading to phase separation [50].

The samples exhibited a reversible phase transition in water from opaque to transparent colorless solution as the temperature dropped, showing similar results to the literature [51]. It was observed that the homopolymer A1 began to whiten at 32 °C, which is in accordance with the value achieved by the previous authors [52]. The cloud point analysis of PNVCL/NVP copolymer samples resulted in an increase in LCST when incorporated with NVP monomer during photopolymerisation. The copolymerisation of PNVCL with NVP and the subsequent increase in LCST have been similarly demonstrated in the literature [14,53].

The copolymers samples containing VAc displayed the opposite trend; the T_cp_ decreased with respect to increasing VAc content. It has been reported that this decrease in T_cp_ is due to the more hydrophobic nature of VAc compared to PNVCL [20]. The work by Kermagoret et al. (2013), in fact, displayed that the T_cp_ could be reduced to as low as 19 °C when incorporating a 45 mol% fraction of VAc.

**Figure 6:**
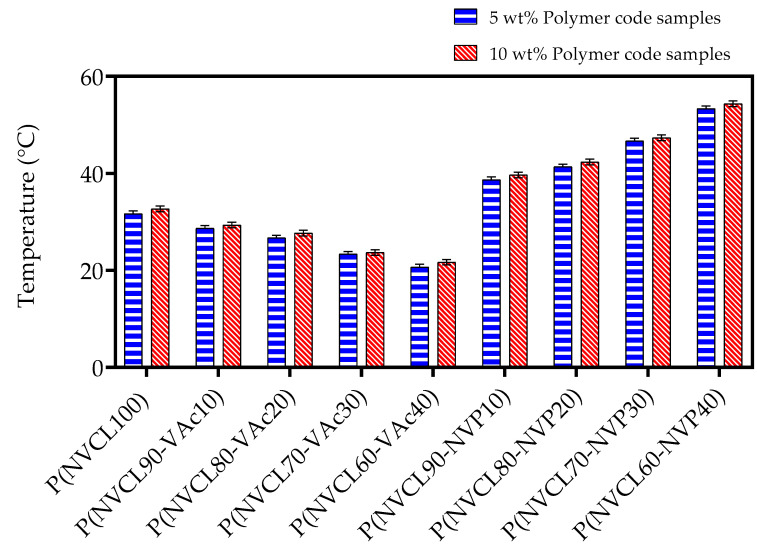
Cloud point analysis of PNVCL, PNVCL/VAc, and PNVCL/NVP for both 5 and 10 wt% polymer samples. Error bars represent the standard error from the mean.

It is evident that the 10 wt% solutions took slightly longer to show signs of turbidity. B1 at 5 wt% concentration began to whiten at 26 °C, whereas the 10 wt% concentration of the same sample clouded at 27 °C. By contrast, the higher the concentration of VAc present, the lower the T_cp_. Overall, the LCST can be increased/decreased by incorporating monomers, which provides flexibility and selectivity for a 4D-printed object when required at different temperatures. Figure 6 displays a bar chart for the 5 and 10 wt% solutions.

#### 3.4.2. UV-Spectroscopy Analysis

**Figure 7:**
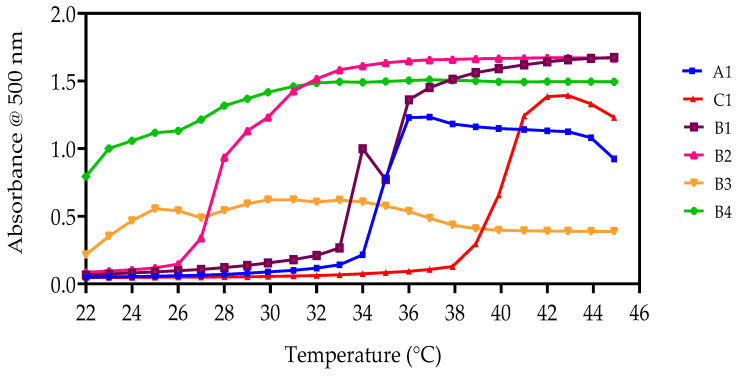
UV Spectrometry illustrating the LCST of the 5 wt% polymer solutions for PNVCL/VAc and PNVCL/NVP samples. (■) PNVCL100, (▲) PNVCL90-NVP10, (■) PNVCL90-VAc10, (▲) PNVCL80-VAc20, (▼) PNVCL70-VAc30, and (◆) PNVCL60-VAc40.

Aqueous solutions of NVCL-based copolymers (5 and 10 wt%) were also investigated to determine the LCST using UV-spectroscopy. Table 5 outlines the results obtained for the 5 and 10 wt% PNVCL, PNVCL/Vac, and PNVCL/NVP aqueous polymer samples. Additionally, Figure 7 illustrates the phase transition behaviour of samples plotted as a function of absorbance versus temperature for each aqueous polymer concentration. All test specimens were quantified over a temperature range of 25 °C to 45 °C. At temperatures below the LCST of the homopolymer, the solution is transparent, whereas above the LCST, the solution is opaque. The transparency at temperatures <LCST is due to H-bond formation between the polymer and water molecules, while at temperatures >LCST, these H-bonds are broken, thereby causing precipitation of the solubilised polymer [54]. The homopolymer PNVCL displayed a LCST of 35 °C, within the range noted in previous observations [34]. As was stated in the aforementioned paper, the stark increase in absorbance at 35 °C denotes that the phase transition had occurred. The LCST of B3 and B4 with 5 wt% aqueous polymer solutions could not be recorded by this technique as the LCST of these formulations was <25 °C. This follows the trend seen whereby increasing the proportions of VAc leads to a decrease in the LCST. Likewise, PNVCL/NVP samples consisting of 20, 30, and 40 wt% concentrations of NVP were not detected by this test method as the LCST was much greater than the range of the machine.

#### 3.4.3. Gelation Determination

**Figure 8:**
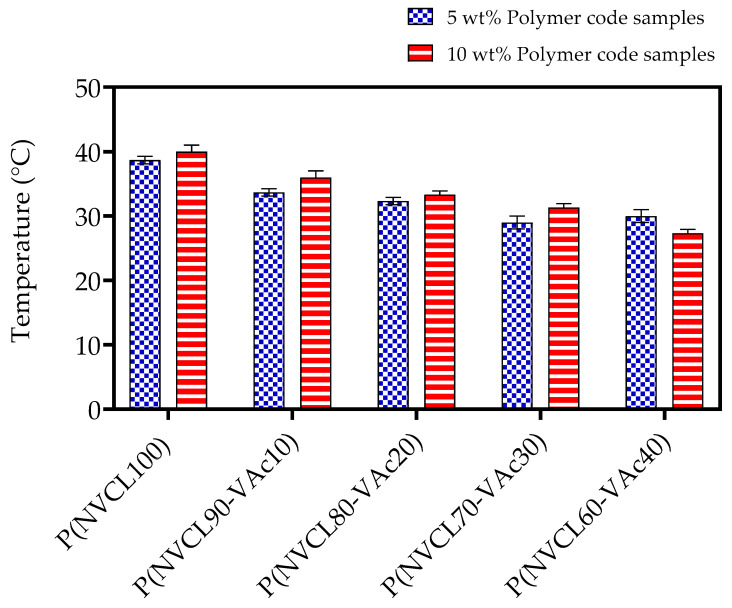
Gelation determination of PNVCL with VAc polymers for both 5 wt% and 10 wt% samples.

**Figure 9:**
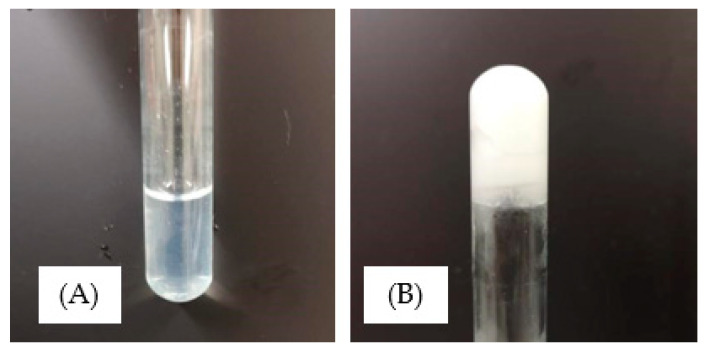
Illustration of the gelation temperature of the ungelled (**A**) and gelled samples (**B**).

The thermo-responsive gelation behaviour of the PNVCL/VAc copolymers were observed by the inverted-tube method. This method determines the sol–gel state of the polymer through examination of the flow/non-flow behaviour of the sample in question in an inverted test tube. Table 6 outlines the gelation results obtained for PNVCL, PNVCL/Vac, and PNVCL/NVP aqueous polymer samples. Figure 8 displays a bar chart of PNVCL with VAc polymers for both 5 and 10 wt% samples while Figure 9 displays a representative visual inspection of the sol–gel transition of the PNVCL-based gels. Both 5 and 10 wt% copolymer solutions displayed a sol–gel transition. The addition of an increasing proportion of VAc into the formulation led to a decrease in the temperature at which the transition occurs. Thermogelling materials have good potential in drug delivery systems (DDSs) as they may act as in situ gel depots, thereby increasing the pharmacokinetics of the contained API [55]. The incorporation of VAc into PNVCL-based hydrogels can be used to modulate the sol–gel transition temperature as shown by the results of this work. The preparation of smart DDSs utilizing negative temperature responsive hydrogels has been proposed wherein API is incorporated directly during the polymerisation process. Such a technique involves mixing API with the thermoresponsive polymer at room temperature prior to injection into the body. Subsequently, the increase in temperature (i.e., from room temperature to physiological temperature) would induce a phase change in the polymer to form a gel. Recently, Andrgie et al. (2020) have developed an injectable gel-forming polymer loaded with ibuprofen to address inflammation and pain during would healing with in vitro studies displaying a reduction in proinflammatory mediators due to the release of API [56].

### 3.5. Swelling Analysis

**Figure 10:**
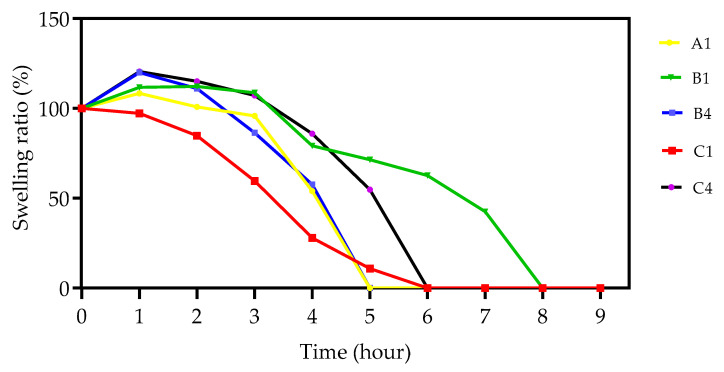
The swelling ratio of PNVCL, PNVCL/VAc, and PNVCL/NVP physically crosslinked samples were conducted at ambient temperature (~22 °C). (●) PNVCL100, (▼) PNVCL90-VAc10, (■) PNVCL60-VAc40, (■) PNVCL90-NVP10, and (●) PNVCL60-NVP40.

One of the most important parameters in relation to controlling the release of API in DDSs is the swelling ratio of the utilised polymers [56]. To examine the effect that incorporating different proportions of monomer had on the copolymer samples swelling capabilities, a static swelling study was conducted at ambient temperature with PNVCL acting as a control sample with graphical results shown in Figure 10. All of the samples had reached their maximum swelling ratio after 1 h, and beyond this point began to gradually reduce in mass and subsequently break down. After 8 h, all samples had lost structural integrity and could no longer be weighed. This rapid dissolution profile of the samples can be attributed to the fact that the study was conducted at a temperature below the LCST of samples as determined by previous studies (Section 3.4.1, Section 3.4.2 and Section 3.4.3). When the surrounding media is maintained below the LCST of the polymer, the dominant interaction is that of H-bonding between the hydrophilic polymer segments compared to that of the interaction between polymer chains and the surrounding water. This in turn leads to the polymeric system absorbing water, which in turn leads to the dissolution of the polymer chains [57,58].

## 4. Conclusions and Future Perspective

In summary, NVCL-based copolymers were successfully polymerised through UV photopolymerisation, as confirmed by FTIR spectroscopy. The LCSTs of the hydrogels were determined by employing three methods: DSC, UV spectroscopy, and cloud point measurements. This study allows for a greater understanding of the LCST phase transition temperature of novel NVCL-based copolymers whose thermal properties were modulated by the inclusion of additional monomer proportions. It was identified that the LCST of PNVCL/NVP increased with increasing concentrations of NVP. By contrast, the LCST of PNVCL/VAc decreased with increasing concentration of VAc. The main focus of this paper is the ability to shift the phase transition temperature of PNVCL to provide flexibility in altering the transition above or below physiological temperature. Further work will be undertaken to investigate the 3D printability of the formulations. Such work is currently underway, and results will be presented in the future.

## Figures and Tables

**Table 1:** Chemical structure of the selected materials.

Material	Chemical Structures
*N*-vinylcaprolactam (NVCL)	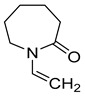
Vinylacetate (VAc)	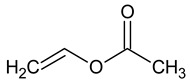
*N*-vinylpyrrolidone (NVP)	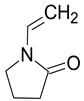
4-(2hydroxyethoxy) phenyl-(2-hydroxy-2-propyl) ketone (Irgacure^®^ 2959)	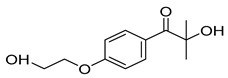

**Table 2:** Code and compositions of copolymerised hydrogels.

ID Code	Formulation	Photoinitiator	Monomer	Comonomers	
		Irgacure^®^ 2959 (wt%)	NVCL (wt%)	NVP (wt%)	VAc (wt%)
A1	P(NVCL100)	0.1	100	--	--
B1	P(NVCL90-VAc10)	0.1	90	--	10
B2	P(NVCL80-VAc20)	0.1	80	--	20
B3	P(NVCL70-VAc30)	0.1	70	--	30
B4	P(NVCL60-VAc40)	0.1	60	--	40
C1	P(NVCL90-NVP10)	0.1	90	10	--
C2	P(NVCL80-NVP20)	0.1	80	20	--
C3	P(NVCL70-NVP30)	0.1	70	30	--
C4	P(NVCL60-NVP40)	0.1	60	40	--

**Table 3:** Characteristic peaks obtained via ATR-FTIR analysis and the interpretation thereof.

Sample	Wavelength (cm^−1^)	Functional Group
Monomers		
NVP	1250	C=O
	1640	C-H
VAc	1730	C=O
NVCL	1656	C=C
Polymerised Samples		
PNVCL	1623	C=O
	2926	C-H
	3440	O-H
PNVCL/VAc	1735	C=O
PNVCL/NVP	1386	C-N-C
	1425	C-N
	1699	C=O

**Table 4:** T_cp_ (°C) results of PNVCL, PNVCL/Vac, and PNVCL/NVP aqueous polymer samples.

ID Code	T_cp_ (°C)	
	5 wt%	10 wt%
A1	32	33
B1	26	27
B2	25	26
B3	23	24
B4	21	22
C1	39	39
C2	41	42
C3	47	48
C4	53	54

**Table 5:** The LCST (°C) results were obtained for the 5 and 10 wt% PNVCL, PNVCL/Vac, and PNVCL/NVP aqueous polymer samples.

ID Code	UV-Spectrometry LCST (°C)	
	5 wt%	10 wt%
A1	35	36
B1	34	34
B2	29	30
C1	36	37

**Table 6:** The gelation (°C) results of the PNVCL, PNVCL/Vac, and PNVCL/NVP aqueous polymer samples.

ID Code	Gelation Temperature (°C)	
	5 wt% Concentration	10 wt% Concentration
A1	39	39
B1	34	35
B2	32	33
B3	30	31
B4	26	27

## Data Availability

Not applicable.
